# Cases of Trichiniasis, and Successful Treatment

**Published:** 1870-03

**Authors:** C. B. Reed

**Affiliations:** Hampshire, Kane County, Illinois


### Chicago Medical Journal.

A MONTHLY RECORD OF

*Medicine, Surgery, and the Collateral Sciences.*

EDITED BY J. ADAMS ALLEN, M.D., LL.D.; AND WALTER HAY, M.D.

VOL. XXVII. — MARCH, 1870. — No. 3.

### Original Communications.

ARTICLE I. — *Cases of Trichiniasis, and Successful Treatment.* By C. B. REED, M.D., *Hampshire, Kane County, Illinois.*

ON the 24th of last December, in the evening, I was called to see a family of Prussians, living on what is here called the Molony farm, situated in the extreme northwest corner of Kane County, and distant about seven miles from my residence.

As I must be brief, for several reasons, I will endeavor to give only what seems to me most important to medical men, of that which I saw, heard, and did.

I found a large two-story wooden dwelling-house, occupied by two families, one in each story. The messenger conducted me to the upper story, where I found four sick persons belonging to the family of Mr. Fritz Martens — Mr. Martens, Mrs. Martens, his wife, a child in the third year, his son, and a woman, fifty-six years old, his mother.

I investigated these cases as far as practicable that evening, renewing the investigation at an early hour on the following morning; and after a very careful and thorough review of all I

could gather, I was forced to believe that these persons were suffering from trichiniasis, and stated the fact to my patients. I also examined the meat used for food by the family, and assured them that it contained large numbers of trichinæ.

I was informed that, some time previously, a hog had been slaughtered for food; that the hams and fattest portions of the meat had been preserved in brine, while all the lean meat was made into a kind of sausage, which is eaten raw. Every one, so far as I could learn, who had eaten of the raw sausage, except two, who afterwards came under my observation as patients, were then sick; but no one eating of the cooked meat only had sickened.

I could learn nothing of the animal that should have excited suspicions of anything wrong.

Curiosity induced me to visit the lower story of the building, where I found four more persons suffering from the same disease, three of whom appeared near the end of the journey of life, and have since died.

Both families had been looked after by a Homœopath, for two weeks. More cannot be said in his favor, as he had, previous to this time (Dec. 25), given them no intimation of the nature of their ailment; but professed to be treating for bilious difficulty. I am sure that up to this date he did not know *what was the matter*.

My announcement of my diagnosis in these cases produced the greatest excitement in the neighborhood, and led to his dismissal, and upon my suggestion, another regular practitioner was called in to attend those in the lower story of the building. I thought that the responsibility of so many serious cases should not all rest upon one physician.

As all these patients appeared so much alike, only a general enumeration of symptoms would be interesting.

Every one complained of a very sore throat, without any swelling or redness, inability to protrude the tongue, which was swollen, with a red tip and edges, and a seeming tendency to the formation of a smooth, glossy strip along the centre; great difficulty in uttering articulate sounds; an entire loss of appetite, with little or no nausea, but very frequent eructations of wind; great thirst; diarrhœa, with very frequent evacuations, except in the case of Mr. Martens' mother, who had not had a stool for a week — the stools appearing like those of typhoid fever, except in the case of Mr. Martens' son, who had large watery stools, reminding one of the operation of epsom salts. There appeared to be an utter inability to digest vegetable food, bread and other vegetable food in the solid form passing without more alteration than would be produced by a thorough soaking in the fluids.

There was a moist skin in all the cases, while some were covered with a profuse perspiration, though the temperature, taken in the axilla, ranged from 102° to 103½° of Fahrenheit. All had an eruption of a peculiar character, reddish in color, and very copious in most of the cases, the spots varying from a mere point to the size of a silver half dollar, and remaining till the patients recovered. There was a generally swollen condition of all the muscles, in most of the cases, and the fascia and sheaths of the muscles contained a large quantity of serous fluid, which gave the limbs a peculiar appearance that I do not remember to have seen elsewhere.

There was not much pain when all the muscles were quiet, though pressure caused pain at any time. Any movement of the muscles of the body and lower limbs was very painful. The muscles of the arms were not so bad as the lower limbs, nor were their movements so much restricted. The pulse was much accelerated; small and wiry where the bowel complaint seemed to take the lead, and not very full in any of the cases. Mr. Martens had pneumonia, and his mother had pneumonia and peritonitis. A constant wakefulness was a very prominent feature in all the cases.

I had no hopes of the recovery of Mr. Martens or his mother, from the first.

I helped Mr. Martens to break his Homœopathic fast with a spoonful of chicken soup, on the ninth day, and I am informed that one of those who died in the lower part of the house had not taken any nutriment for fourteen days.

Mr. Martens died on the 27th, in the morning, and his mother died on the 2nd of January, following. Nothing was done for Mr. Martens beyond an attempt to sustain the strength, and the administration of aquæ chlorinii a few hours.

His mother took six grains a day of carbolic acid, for four days, which seemed somewhat to modify the disease; and she also took frequent doses of castor oil and turpentine, for the purpose of thoroughly clearing out the alimentary canal.

After her death a portion of muscle was examined microscopically, and found to contain a large number of living trichinæ—a very sufficient proof that the quantity of the acid given was not enough to kill the worms outside of the alimentary canal.

Mr. Martens' wife and child were the last persons on the premises who came down with the disease. They were as sick as any of the others, and as treatment has seemed to be successful, in their cases, I have no doubt it will be interesting, and therefore give its most important features:

| | | |
|---|---|---|
| ℞ | Carbolic Acid, - - - - - | grs. xij; |
| | Oil of Turpentine, - - - - - | f ʒ j; |
| | Tr. of Opium, - - - - - | gtt. xc; |
| | Gum Arabic, - - - - - - | ʒ ix; |
| | Loaf Sugar, - - - - - - | ℥ j; |

Water to make f ℥ vj of the mixture, of which f ℥ ss was to be given every two hours.

Two grains of quinine were given every eight hours, and a teaspoonful of alcohol, flavored with sassafras, and properly diluted, was given every six hours. A full dose of a mixture of castor oil and oil of turpentine was given every day, for a few days, as a cathartic.

When the emulsion had been continued about ten days, she became anasarcous, and it was omitted, and a diuretic mixture substituted.

She was in the sixth month of pregnancy, and had some œdema of the limbs before she became trichinosed. She miscarried January 10th, and all medicine was laid aside except two grains of quinine three times a day.

On the 16th I found the swelling of the limbs and dropsical effusion nearly gone, the soreness was not any longer apparent; and she did, in spite of my remonstrances, get up and go about the house. At this time she resumed her ordinary diet, which had previously been restricted to a very generous supply of animal food in the fluid form.

The only medicine directed at this time was ten drops of tincture of muriate of iron to be taken three times a day.

On the 19th, she was able to attend to her duties about the house, and was removed to the residence of friends at a distance.

It is proper that I should here acknowledge my obligations to Drs. Hagar and Stull of Marengo for timely and valuable suggestions in the case of Mrs. Martens, though they were not employed. The boy, son of Mr. Martens, who was in his third year, was treated with an emulsion containing somewhat more than the proportionate quantities of acid and turpentine, and a small quantity of paregoric instead of laudanum, with daily cathartic doses of castor oil and oil of turpentine; and a large allowance of animal food in the fluid form. His recovery was much more rapid than his mother's.

On the 29th of December, Mr. Charles Rathka and wife, who live about a mile from the Molony farm, applied to me for advice, they having eaten freely of the raw sausage while visiting at the Molony farm on the 20th. Mr. Rathka showed no other signs of the disease than very slight indigestion. Mrs. Rathka had sore throat, as she called it, indigestion, frequent eructations of wind, loss of appetite, a constant very disagreeable feeling in the stomach, some soreness of the muscles, an accelerated circulation, an increased heat of the surface, and a coated tongue, red at the tip and edges. I gave each one a cathartic of castor oil and oil of turpentine, with half a grain of carbolic acid, to be followed by one grain of carbolic acid dissolved in water every six hours. On the 30th, not perceiving any improvement, I gave both of them a cathartic of castor oil and oil of turpentine, to be followed by one grain of carbolic acid dissolved in mucilage of gum Arabic every hour, to be continued until both had taken twelve grains of the acid.

Treatment was then discontinued.

On the 2nd of January Mr. Rathka was well, and his wife had only a slight stiffness about one ankle, which soon subsided; and they have both remained well up to this time.

Results may be stated as follows: two deaths and three recoveries, (not counting Mr. Rathka); though as no hopes were entertained of the recovery of Mr. Martens and his mother from the first time that I saw them, I do not think their cases should be counted in summing up results.

I am indebted to the politeness of my friend and former preceptor, Dr. Joseph Tefft, of Elgin, for a microscopic examination of the portions of meat and muscle previously referred to, and for an estimate of the number of trichinæ to the square inch in a portion of ham that I sent him, which according to his estimate was about eight thousand.

The Doctor also has my thanks for the opportunity he gave me of seeing the worms alive with his excellent instrument.

